# β-Glucuronidase at the Microbiota—Host Interface: Dual Regulatory Roles and Precision Modulation by Natural Products

**DOI:** 10.3390/molecules31040601

**Published:** 2026-02-09

**Authors:** Jialu Shen, Shuai Xu, Qingyu Zhao, Junmin Zhang, Huiyan Zhang

**Affiliations:** State Key Laboratory of Animal Nutrition and Feeding, Institute of Animal Sciences, Chinese Academy of Agricultural Sciences, Beijing 100193, China

**Keywords:** β-glucuronidase, gut microbiota, structural classification, natural products, GUS inhibitors

## Abstract

Gut microbial β-glucuronidase (GUS) plays a pivotal role at the microbiota—host interface by hydrolyzing glucuronide conjugates, thereby influencing xenobiotic metabolism, enterohepatic circulation, and systemic homeostasis. Dysregulated GUS activity has been increasingly linked to adverse health outcomes, including drug-induced toxicity, inflammation, and cancer. However, current literature often overlooks the enzyme’s dual role in maintaining physiological balance and promoting disease progression, as well as the multidimensional ways in which natural products interact with GUS. This work reviews recent advances in GUS research, emphasizing its structural diversity, functional complexity, and regulatory impact on host health. It also highlights the potential of natural products as precision modulators of GUS activity, capable of direct enzyme inhibition or indirect modulation through reshaping the gut microbiota. These mechanisms collectively influence drug efficacy, toxicity, and the systemic availability of endogenous metabolites. By integrating structural, pharmacological, and microbiological perspectives, this work provides a theoretical foundation for the development of microbiota-targeted therapies centered on GUS. Such approaches may support the rational design of natural product-derived inhibitors and promote their application in disease models, ultimately advancing personalized therapeutic strategies.

## 1. Introduction

The human gut microbiota, comprising trillions of microorganisms, plays essential roles in host physiological processes, including metabolism [1] and immune regulation [2]. The intricate relationship between this microbial ecosystem and host health has become a major research focus [3]. Accumulating evidence demonstrates that the gut microbiota modulates chemotherapeutic drug efficacy or toxicity through diverse mechanisms, such as immune modulation [4], enzymatic biotransformation [5], and alterations in drug pharmacokinetic profiles [6]. It can thus be inferred that not only the gut microbiota themselves but also their secreted functional enzymes are key mediators of the microbiota–chemotherapy interactions.

Among these enzymes, gut microbial β-glucuronidase (GUS), produced by a broad range of gut microorganisms, has attracted substantial research interest. As a member of the glycoside hydrolase family, GUS catalyzes the hydrolysis of glucuronide moieties from both small molecules and complex carbohydrates [7]. GUS is a central microbial biocatalyst that modulates the activity and availability of a wide range of glucuronidated compounds. By reversing glucuronidation in the intestine, GUS influences both host metabolic homeostasis and the biological fate of exogenous substances such as drugs and toxins [8]. Given its broad regulatory significance, microbial GUS has emerged as an important mediator linking gut microbiota function to systemic physiology and disease [9].

The influence of GUS on host physiology exhibits a dual nature, manifesting as a classic “double-edged sword” effect. On one hand, GUS facilitates the systemic metabolism of glycosides, thereby promoting the bioactivity of released aglycones and maintaining homeostasis of endogenous substances [10,11]. On the other hand, aberrant GUS activity has been implicated in diverse pathological conditions. For instance, in endometrial and breast cancers [12,13], gut microbial GUS enhances the reabsorption and reactivation of estrogens, elevating circulating estrogen levels that subsequently drive tumor progression. Moreover, specific microbial GUS isoforms can reactivate detoxified chemotherapeutic drugs in the intestinal lumen, causing local drug accumulation and severe gastrointestinal toxicity. To overcome this clinical limitation, strategies focused on developing novel inhibitors or structurally optimizing existing compounds to target GUS have been pursued. While these approaches have proven effective in alleviating GUS-mediated drug side effects in mouse models, their clinical translation faces substantial barriers, including lengthy development timelines, high costs, and safety profiles that necessitate further comprehensive evaluation [14].

Accordingly, natural products derived from diverse biological sources, including plants, bacteria, and fungi, have emerged as promising candidates for targeting GUS, owing to their diverse biological activities, broad therapeutic windows, and potential for multi-target, synergistic regulation [15]. Leveraging GUS enzymes from representative gut microbes such as *E. coli*, extensive screenings of natural products have uncovered numerous inhibitory compounds. Concurrently, in vivo studies on structurally characterized natural compounds have validated their GUS-modulating effects, demonstrating that natural products can regulate GUS activity through both direct enzymatic inhibition and indirect remodeling of GUS-producing microbial populations. Collectively, these efforts are expanding the molecular repertoire of GUS inhibitors and establishing a solid conceptual and theoretical foundation for the development of GUS-targeted intervention strategies [16].

Current reviews primarily emphasize the mechanistic roles of GUS in tumorigenesis and the modulation of anticancer drug responses, as well as the development of synthetic GUS inhibitors. However, they largely overlook a systematic synthesis of GUS’s dual regulatory functions as a critical interface in host–microbiota interactions, particularly its roles in maintaining metabolic homeostasis through the regulation of endogenous substrates—such as bile acids and steroid hormones. Moreover, existing literature lacks comprehensive coverage of the multifaceted interactions between GUS and natural products, which offer inherent advantages in structural complexity and multi-target regulation.

To address these gaps, this review focuses on gut microbiota-derived GUS, synthesizing its key regulatory functions in health and disease. We comprehensively examine both the direct and indirect mechanisms underlying GUS–natural product interactions and summarize current evidence regarding their synergistic contributions to systemic homeostasis and disease modulation. By filling these critical gaps, this work aims to provide a theoretical framework for the targeted screening of natural GUS modulators and for advancing mechanistic understanding, while also offering new perspectives and scientific rationale for innovative disease prevention and therapeutic strategies centered on the gut microbiota–GUS axis.

## 2. GUS Overview

### 2.1. GUS Structure and Classification

Since its first identification in animal tissues by Masamune et al. in the 1940s, GUS has emerged as a pivotal enzymatic mediator of glucuronide deconjugation, thereby counteracting glucuronidation through deconjugation of glucuronide metabolites [17]. A major milestone was achieved in 1996, when Jain et al. resolved the first crystal structure of human GUS at 2.6 Å resolution [18]. The enzyme forms a homotetramer exhibiting approximate dihedral symmetry. Each monomer consists of three structural domains: a jelly-roll barrel, an immunoglobulin-like domain, and a TIM barrel fold. Notably, a hairpin loop formed by residues 179–204 resembles a putative lysosomal targeting motif observed in cathepsin proteases, suggesting a conserved mechanism underlying lysosomal localization. The active site is located within a deep cleft at the dimer interface, with Glu451 and Glu540 identified as key catalytic residues. This structural insight not only clarified the molecular basis of mutations causing mucopolysaccharidosis type VII but also provided a structural framework for resolving GUS homologs from diverse biological sources, including microbial and mammalian tissues.

As research on microbiota—host interactions has progressed, microbial metabolites have been increasingly recognized as critical mediators, with gut microbial GUS becoming one of the most extensively studied examples. A significant advancement occurred in 2010, when Wallace et al. resolved the crystal structure of *Escherichia coli* β-glucuronidase (EcGUS) at 2.5 Å resolution [19]. The structure exhibits substantial similarity to human β-glucuronidase, displaying a conserved tertiary fold and a tetrameric assembly. The N-terminal segment (residues 1–180) adopts a fold resembling the carbohydrate-binding domain characteristic of the GH2 glycoside hydrolase family, whereas the C-terminal region (residues 274–603) forms an α/β barrel that harbors the key catalytic residues Glu413 and Glu504 [20]. An immunoglobulin-like β-sandwich domain connects the N- and C-terminal regions, a structural feature conserved across the GH2 family.

Structural comparison between human and gut microbial GUS proteins reveals high similarity between the *Escherichia coli* enzyme and human β-glucuronidase, with a backbone Cα RMSD of 1.4 Å across 565 aligned residues and approximately 45% sequence identity. Notably, EcGUS contains a 17-residue insertion, termed the “bacterial loop,” which is absent in the human enzyme. This structural element contributes to the formation of the active site within its own monomer and also interacts with the corresponding loop from an adjacent monomer to complete the active—site architecture. Analysis of 123 gut microbial GUS sequences from the Human Microbiome Project revealed that 98% of these enzymes retain at least the N-terminal portion of the bacterial loop, while 91% (110 sequences) preserve the key inhibitor-binding residues corresponding to positions 361 and 365 in EcGUS. These findings indicate that the bacterial loop is highly conserved among gut microbial GUS enzymes, providing a structural basis for the development of inhibitors that selectively target microbial GUS activity.

Although over 800 gut microbial GUS enzymes have been reported to date, with highly conserved catalytic residues in their active sites, the bacterial loops adjacent to these sites exhibit substantial diversity in both amino acid composition and structural conformation. Through systematic screening of the Human Microbiome Project database, Pollet et al. constructed the first comprehensive landscape of human gut GUS enzymes, identifying 3013 sequences that were subsequently curated into 279 non-redundant unique proteins [21]. These enzymes exhibit strong conservation in both catalytic residues and the core α/β hydrolase fold. Based on variations in loop length and sequence near the active site, GUS enzymes were classified into six major structural categories: Loop 1 (L1), Mini-Loop 1 (mL1), Loop 2 (L2), Mini-Loop 2 (mL2), Mini-Loop 1,2 (mL1,2), and No Loop (NL). An additional category, termed “No Coverage (NC),” comprises sequences with undetermined loop regions due to ambiguity in translation start or stop site annotation; these have been collectively referred to as the “dark matter” of the dataset.

To evaluate the broader applicability of this classification framework, Creekmore et al. constructed a “mouse GUSome” using high quality mouse gut metagenomic datasets, identifying 444 unique GUS proteins. Despite sequence level divergence, mouse and human gut microbial GUS enzymes retain conserved core functions and can be classified into the same six major structural categories [22], supporting the cross-species relevance of this framework. In addition, structurally distinct GUS variants have also been reported. One example is Fp2GUS, isolated by Pellock et al. from the human gut bacterium *Faecalibacterium prausnitzii* L2–6. The purified protein appeared yellow, and absorption spectroscopy confirmed it as a flavin-binding enzyme. Structural analysis revealed that flavin mononucleotide (FMN) binds within a distinct pocket located ~30 Å from the catalytic center, suggesting a role in structural stabilization or substrate affinity modulation rather than direct catalytic participation. Bioinformatic analyses further identified 14 FMN-binding GUS homologs in the human gut microbiome, indicating that such regulatory forms of GUS are relatively prevalent among commensal microbes. Although FMN binding does not affect catalytic activity, it may influence substrate affinity, implying that FMN-associated GUS variants could fulfill more complex regulatory roles in intestinal metabolism—an aspect warranting further investigation [23].

More recently, several studies have proposed designating the N-terminal loop (NTL) as an additional, eighth structural category of GUS [24]. However, given that this group lacks a canonical Loop 1 structure, it can reasonably be classified within the existing NL type. Structural evidence suggests that NTLs from adjacent subunits within the tetrameric assembly collectively form a functional architecture analogous to Loop 1 [25]. Therefore, a separate classification is deemed unnecessary.

### 2.2. Diversity and Functional Specialization of GUS

Gut microbial GUS is ubiquitously present within the gastrointestinal microbiota of most mammals, including both humans and mice [26]. However, despite this wide-spread functional presence, GUS genes are confined to a relatively limited subset of intestinal bacterial species. Metagenomic assembly analyses indicate that approxi-mately 25–26% of gut bacterial genomes encode GUS [27], and among the thousands of taxa detected in the human gut, at least 50 species have been identified as GUS carriers [11]. These organisms are predominantly affiliated with the phyla *Bacteroidota* and *Bacillota* [13,28,29], whereas GUS-positive genomes remain rare in *Pseudomonadota* and *Verrucomicrobiota*, accounting for less than 2% [21,22]. Together, these inter-species differences underscore the host-dependent variability of microbiome-encoded GUS repertoires and suggest that microbial community architecture may shape glucuronide processing capacity and xenobiotic metabolism across mammals. Although GUS-encoding species have occasionally been reported in *Verrucomicrobiota*, their abundance remains low, and functional characterization is still limited [29]. These bacterial taxa encode a wide array of carbohydrate-active enzymes (CAZymes), and the vast majority of gut microbial GUS enzymes are classified within glycoside hydrolase family 2 (GH2) [30].

However, notable exceptions to this classification have been identified. Cartmell et al. identified an arabinogalactan β-1,6-glucuronidase (BT3677) from the human gut bacterium *Bacteroides thetaiotaomicron*, which belongs to glycoside hydrolase family 154 (GH154)—the only biochemically characterized member of this family to date. Its enzymatic activity requires prior removal of terminal rhamnose residues by a GH145 family α-L-rhamnosidase (BT3686) [31]. GUS-like activity has also been reported in other glycoside hydrolase families, including GH1, GH30, and GH79 [32]. It is important to note, however, that these enzymes predominantly originate from mammalian tissues, fungi, or soil microorganisms and play minimal roles in the intestinal enterohepatic circulation of glucuronidated compounds [33,34,35,36,37]. Their functional roles appear to be specialized for hydrolyzing endogenous steroid β-glucuronide conjugates or facilitating the breakdown of specific flavonoid aglycones [33,34].

Distinct bacterial phyla exhibit clear preferences in the distribution of GUS structural types. For example, *Bacillota* encode diverse GUS variants, including L1, mL1, L2, NL, and FMN-binding types, whereas *Verrucomicrobiota* appear to encode exclusively the mL1,2 type (Table 1). By analyzing N-terminal signal peptides, Pollet et al. further linked GUS structural classification and subcellular localization to bacterial phylogeny, revealing a “spatial division of labor” among GUS types in substrate utilization [21]. Their findings demonstrated that transmembrane localization of GUS enzymes exhibits structural-class and phylum specific patterns (Table 1): L1-type GUS enzymes lack signal peptides and are localized intracellularly; L2, mL2, and mL1,2 types possess signal peptides and are targeted to the periplasmic space. The localization of mL1 and NL types appears phylum dependent: all *Bacillota*-derived variants lack signal peptides, whereas those from *Bacteroidota* consistently contain them. This distribution pattern suggests functional spatial partitioning: periplasmic GUS may primarily act on large polysaccharide substrates, whereas intracellular GUS is more likely to hydrolyze small molecule glucuronides that can diffuse across cell membranes. This work not only advances mechanistic understanding of intestinal GUS function, but also integrates bacterial phylogeny, enzyme localization, and metabolic activity—thus providing a theoretical framework for interpreting microbial metabolism through the lens of ecological niche specialization.

## 3. GUS Mediates Host Health and Disease

In living organisms, drugs, toxins, and endogenous substances, including estrogens, bilirubin, and serotonin, undergo biotransformation through multiple metabolic pathways [38]. This process is conventionally divided into two major phases. Phase I metabolism introduces or exposes functional groups via oxidation, reduction, or hydrolysis. Phase II metabolism involves conjugation with hydrophilic moieties, such as glucuronic acid, glutathione, or sulfate, producing more polar metabolites that are readily excreted [39,40]. A portion of the resulting glucuronide conjugates is transported to the intestinal lumen via bile, where they can be hydrolyzed by gut microbial GUS enzymes. Through this specific hydrolytic activity, GUS directly participates in the enterohepatic circulation of numerous endogenous compounds and xenobiotics, thereby modulating their metabolic flux and contributing to systemic homeostasis [41,42]. However, dysregulated or aberrantly elevated GUS activity can disrupt enterohepatic cycling, promote the reaccumulation of toxic metabolites, and disturb the balance of endogenous substances. These perturbations have been associated with a range of pathological conditions, including chemotherapy-induced intestinal toxicity [43], primary intrahepatic choledocholithiasis [44], hormone-related cancers [12], and intestinal disorders such as irritable bowel syndrome and inflammatory bowel disease [45].

### 3.1. A Central Role for GUS in Systemic Homeostasis

**Figure 1:**
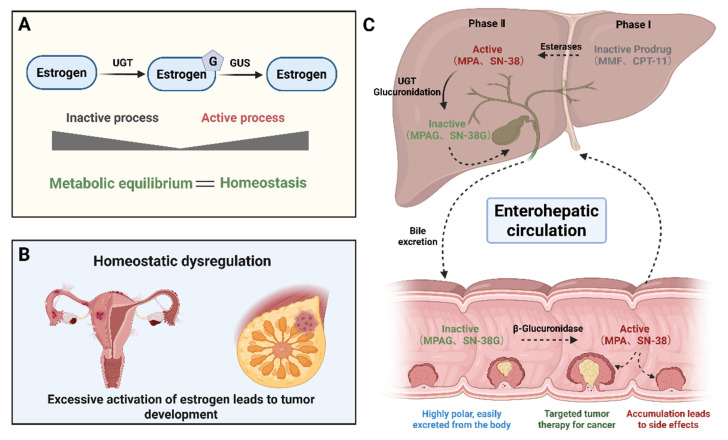
GUS-mediated biotransformation of endogenous and exogenous substances. (This figure was created using BioRender (https://BioRender.com/uc7qj2r). (**A**) Role of GUS as a central regulator in estrogen-related homeostasis. (**B**) Homeostatic dysregulation driven by aberrant GUS activation. (**C**) Contribution of GUS to gastrointestinal toxicity induced by exogenous drugs (MMF and CPT-11). In the schematic, pink areas represent intact intestinal epithelium, yellow regions indicate tumor foci, and dark red regions denote drug-induced ulcerative damage.

Among endogenous substrates, estrogens represent one of the most biologically and clinically significant targets of microbial GUS activity. Estrogens are primarily synthesized in the ovaries, with additional contributions from the adrenal glands and peripheral tissues through aromatization of androgen precursors, while their production is systemically regulated by the hypothalamic–pituitary–gonadal axis. After exerting biological effects in target tissues, circulating estrogens undergo metabolic inactivation predominantly in the liver, generating estrogen–glucuronide conjugates with reduced biological activity and increased water solubility. These conjugated metabolites are efficiently excreted into the intestine via bile, thereby becoming accessible substrates for gut microbial enzymes. Within the intestinal lumen, GUS selectively deconjugates estrogen–glucuronide metabolites, regenerating free, bioactive estrogens that can be reabsorbed across the intestinal epithelium and returned to the systemic circulation. Through this microbiota-dependent process, GUS fine-tunes systemic estrogen homeostasis and contributes to endocrine balance [46] (Figure 1A). Through this pathway, GUS activity has been shown to alleviate menopausal symptoms, including hot flashes, vaginal dryness, and mood disturbances. It also supports estrogen’s protective effects on bone density and vascular function, potentially reducing the risk of osteoporosis, cardiovascular disease, and urogenital atrophy [47]. However, the role of GUS in estrogen signaling also extends to disease states. Aberrant GUS activity has been implicated in the pathogenesis of hormone-dependent cancers such as breast, endometrial, and ovarian cancers [1,48] (Figure 1B). In addition, in endometriosis, gut dysbiosis can upregulate GUS expression, leading to excessive deconjugation of estrogens and a subsequent increase in circulating estradiol. This creates a pathological feed-forward loop that exacerbates disease progression [49].

Beyond estrogen, GUS functions as a master regulator of multiple endogenous compounds through its reciprocal relationship with hepatic UGT enzymes. A representative example is bilirubin, a cytotoxic heme catabolite. In the liver, UGT catalyzes the conjugation of bilirubin for biliary excretion, after which intestinal GUS hydrolyzes the conjugates back into free, insoluble bilirubin [50]. This unconjugated form is subsequently converted by gut microbiota into urobilinogen, completing an enterohepatic cycle in which 10–20% is reabsorbed and either resecreted by the liver or released into systemic circulation for renal elimination [51].

The regulatory reach of GUS extends to a broad array of endogenous molecules, including bile acids, monoamine neurotransmitters, thyroid hormones, steroid hormones, melatonin, vitamins, and glycosaminoglycans [52,53,54,55,56,57]. By hydrolyzing their glucuronide conjugates, GUS regenerates bioactive aglycones, thereby enhancing both their enterohepatic recirculation and systemic bioavailability. Through this multifaceted activity, GUS serves as a central modulator of systemic homeostasis, tightly linking gut microbial function to core physiological processes such as lipid metabolism, gut–brain axis signaling, reproductive regulation, and maintenance of tissue integrity.

### 3.2. GUS Mediated Drug Metabolism and Toxicity

Glucuronidation represents a major metabolic pathway for drug biotransformation in vivo, substantially affecting the pharmacological and toxicological profiles of compounds by modifying their physicochemical properties. This process typically converts parent drugs into more hydrophilic glucuronide conjugates, thereby facilitating excretion and generally promoting detoxification. However, glucuronidation is not exclusively a detoxification mechanism; in certain cases, it can generate metabolites with complex or even enhanced biological activity. This dual nature is particularly evident in the metabolism of carboxylic acid-containing drugs (CADs), whose glucuronide conjugates have been closely linked to adverse toxicological outcomes.

CADs represent a class of therapeutic agents that are highly susceptible to glucuronidation. This category includes widely used nonsteroidal anti-inflammatory drugs (NSAIDs, e.g., diclofenac, ibuprofen), opioid analgesics (e.g., morphine, codeine), antineoplastic agents such as irinotecan, statins, and other compounds that generate carboxylic acid metabolites during biotransformation [58]. Although glucuronidation generally promotes clearance, CAD-derived acyl glucuronides are chemically reactive and can undergo intramolecular rearrangement or form covalent adducts with cellular proteins and nucleic acids, thereby contributing to adverse toxicological outcomes [59].

The glucuronide conjugation of CADs is further complicated by their tight coupling to enterohepatic circulation. Following hepatic uptake via organic anion transporters, CADs undergo UGT-catalyzed glucuronidation and are subsequently secreted into the bile, delivering acyl glucuronide conjugates to the intestinal lumen. In the gut, microbial GUS hydrolyzes these conjugates, regenerating the parent compound, which is reabsorbed via the portal circulation and returned to the liver, effectively prolonging systemic and hepatic exposure [60]. Dysregulation of this transport system can alter drug distribution patterns and promote excessive formation and intracellular accumulation of reactive acyl glucuronide metabolites [61]. Once these reactive species surpass a toxicological threshold, they may elicit idiosyncratic adverse effects. A systematic analysis of the DrugBank database has identified more than 100 pharmaceuticals whose metabolic pathways involve GUS, further underscoring the enzyme’s broad role in regulating CAD disposition [62].

Mycophenolate mofetil (MMF) and irinotecan (CPT-11) serve as typical clinical examples of how microbial GUS-dependent intestinal processing may compromise therapeutic efficacy by inducing gastrointestinal toxicity (Figure 1C). Widely used in organ transplantation, MMF has been shown to improve both graft and patient survival. However, its clinical utility is often restricted by dose-limiting gastrointestinal toxicity, which may necessitate dose reduction or discontinuation, thereby increasing the risk of graft rejection.

This toxicity is primarily attributed to enterohepatic circulation [63]. Following administration, MMF is hydrolyzed into the active form, mycophenolic acid (MPA), which undergoes extensive hepatic glucuronidation to form mycophenolic acid glucuronide (MPAG) and a minor acyl-glucuronide. Approximately 10% of these conjugates are excreted into the intestine via biliary transport, where they are hydrolyzed by gut microbial GUS to release free MPA, thereby increasing local intestinal exposure. Elevated luminal MPA levels have been associated with sustained gastrointestinal injury, potentially through multiple mechanisms, induction of mucosal inflammation, and disruption of barrier integrity [64].

Notably, CPT-11 exhibits a metabolic activation and toxicity pathway similar to that of MMF. Its characteristic delayed-onset diarrhea results from local accumulation of the active metabolite 7-Ethyl-10-hydroxycamptothecin (SN-38) in the intestinal epithelium, a process critically dependent on gut microbial GUS [65]. CPT-11 is first converted by hepatic carboxylesterases to SN-38, a potent topoisomerase I inhibitor. SN-38 is then inactivated via UGT1A1-mediated glucuronidation to form SN-38 glucuronide (SN-38G), which is secreted into the bile. Upon reaching the intestinal lumen, SN-38G is hydrolyzed by GUS, regenerating high local concentrations of cytotoxic SN-38. This metabolite induces DNA damage and epithelial apoptosis and elicits a robust inflammatory response, collectively contributing to mucosal injury. A fraction of the liberated SN-38 is reabsorbed into the portal circulation, returned to the liver for re-glucuronidation, and secreted again via bile. This self-perpetuating “hydrolysis–reactivation–reabsorption” cycle continually increases intestinal SN-38 exposure, ultimately resulting in the severe gastrointestinal toxicity observed in 80–90% of treated patients [66].

## 4. Modulating the GUS–Microbiota Axis with Natural Products

### 4.1. Inhibitors Derived from Natural Products Targeting GUS

**Figure 2:**
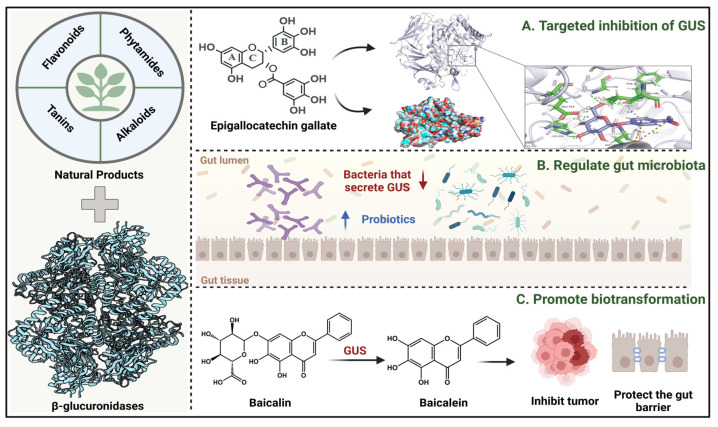
Multidimensional interactions between natural products and GUS (this figure was created using BioRender (https://BioRender.com/oi778gm). (**A**) Direct inhibition of GUS enzymatic activity by natural products. (**B**) Indirect regulation of GUS through natural product-mediated remodeling of gut microbiota. (**C**) GUS-mediated biotransformation of natural products contributing to physiological benefits.

GUS inhibitors are broadly categorized into synthetic and natural product-derived compounds [67]. Although synthetic inhibitors are more commonly utilized, their development is limited by complex synthetic procedures, high production costs, and safety concerns. In contrast, natural products-secondary metabolites derived from biological sources generally demonstrate higher biocompatibility and improved safety profiles, making them a promising direction for GUS-targeted inhibitor development. This has prompted increased interest in the molecular interactions between natural products and gut microbial GUS (Figure 2). The bioactivity of natural metabolites is primarily determined by key functional groups, such as phenolic hydroxyl moieties, conjugated double-bond systems, heterocyclic frameworks, and glycosidic linkages, which collectively influence redox behavior, GUS-binding affinity, and enzyme modulatory capacity toward gut microbial GUS [68]. Given the substantial volume of research in this area, this section consolidates key advances from the past five years, with a focus on compounds that elucidate critical structure–activity relationships (Table 2).

#### 4.1.1. Flavonoid Inhibitors

An increasing number of studies have focused on the direct inhibitory effects of natural products on GUS (Table 2). Among these, flavonoids represent the most extensively investigated class of natural product-derived GUS inhibitors, with a diverse botanical distribution spanning *Ginkgo biloba* [75], *Alhagi graecorum* [69], *Selaginella tamariscina* [70], and *Centaurea scoparia* [74].

Representative flavonoids exhibit distinct inhibitory mechanisms and potency profiles. Myricetin, isolated from *Alhagi graecorum*, functions as a non-competitive inhibitor of EcGUS with an IC_50_ of 3.95 ± 0.04 μM. Its mechanism involves binding to key catalytic residues, including Glu504 and Glu413, as well as Trp549 [69]. In contrast, azaleatin—derived from *Centaurea scoparia*—is a more potent non-competitive EcGUS inhibitor (IC_50_ = 0.57 ± 0.04 μM). It shares a homologous binding site with epigallocatechin gallate (EGCG), engaging residues Phe448 and Glu413, and has been employed for targeted GUS modulation in tumor microenvironments [73]. Amentoflavone displays unique, substrate-dependent inhibition: it acts as a mixed-type inhibitor against the substrate DDAOG (IC_50_ = 0.62 ± 0.072 μM) but as a competitive inhibitor against SN-38 glucuronide (IC_50_ = 0.49 ± 0.03 μM). This dual behavior is governed by specific interactions with EcGUS residues Leu361, Ile363, and Glu413 [70].

#### 4.1.2. Natural Amide Inhibitors

Natural Amide Inhibitors have been primarily isolated from *Piper longum*. These compounds share a common structural scaffold consisting of a piperidine or isobutylamide core linked to side chains that typically contain 2–3 conjugated double bonds in the 2E,4E configuration. The stereochemistry and chain length of these conjugated moieties critically influence their inhibitory activity [77]. Representative compounds within this class exhibit distinct inhibitory mechanisms. Piperlongumamide F functions as a non-competitive inhibitor of EcGUS (IC_50_ = 4.9 ± 0.9 μM), binding to an allosteric site formed by residues Trp340 and Ala316. In contrast, N-isobutyl-(2E,4E)-undecadienamide acts as an uncompetitive inhibitor (IC_50_ = 5.8 ± 1.2 μM), interacting with the enzyme–substrate complex through hydrophobic contacts involving residues Tyr160, Leu361, and Tyr472.

Beyond *Piper* species, structurally more complex phenylpropanoid-derived alkamides have also been reported from other medicinal plants. Grossamide is isolated from Grossheimia schtschukiniana (0.1–0.15% dry weight) and serves as a potent non-competitive inhibitor of EcGUS (IC_50_ = 0.73 ± 0.03 μM). Its activity is mediated by combined electrostatic and hydrophobic interactions involving residues such as Glu413, Glu504, and Arg562. Related analogs further support the inhibitory relevance of this scaffold: grossamide-K retains strong non-competitive inhibition (IC_50_ = 1.24 ± 0.06 μM) with contacts spanning Ser159, Asn358, Leu561, and Arg562, whereas the simpler amide N-feruloyltyramine exhibits weaker activity (IC_50_ = 12.16 ± 0.7 μM). Together, these findings highlight natural amides as emerging GUS inhibitors and suggest that structural optimization of the amide framework may further enhance potency [72].

#### 4.1.3. Tannin Inhibitors

A representative tannin-derived GUS inhibitor is 1,2,3,4,6-penta-O-galloyl-β-D-glucopyranose (PGG), which has been isolated from *Rhodiola crenulata* [78]. The molecule consists of a glucose core fully esterified with galloyl moieties at all hydroxyl groups (C1-C6). This poly-galloylated structure confers both potent non-competitive inhibition across various GUS enzymes and pronounced species selectivity. PGG binds at a non-catalytic site on EcGUS, forming a network of 12 hydrogen bonds. It exhibits particularly high potency against *Clostridium perfringens* GUS (*Cp*GUS), with an IC_50_ value of 0.19 ± 0.001 μM, an effect primarily mediated through hydrogen-bonding interactions with the enzyme’s Loop-1 region.

#### 4.1.4. Catechin and Theaflavin Inhibitors

Inhibitors in this class are predominantly derived from black tea, with catechin gallate and theaflavin-3′-gallate serving as representative compounds. Both exhibit mixed-type inhibition against EcGUS, although they differ substantially in potency—a distinction primarily attributed to differences in molecular complexity.

Catechin gallate, which possesses a monomeric structure containing a single galloyl group, inhibits EcGUS with an IC_50_ of 1.48 μM. Its inhibitory activity is mediated through hydrogen bonding between the galloyl hydroxyl group and Glu413, along with hydrophobic interactions between its benzene ring and residues Ser360 and Ile560. In comparison, the more structurally complex theaflavin-3′-monogallate displays stronger inhibition (IC_50_ = 1.03 μM), engaging residues Leu359, Asn369, and Arg417. The related compound theaflavin-3,3′-digallate also exhibits mixed-type inhibition (IC_50_ = 1.41 μM), with binding contacts overlapping those of catechin gallate, including Ser360, Glu413, and Ile560 [79].

#### 4.1.5. Other Inhibitors

Plants represent the largest natural product reservoir, and among these, alkaloids, phenylpropanoids, terpenoids, and quinones have also been reported as effective GUS inhibitors. Among them, alkaloid angustine has been identified as a inhibitor of EcGUS, exhibiting non-competitive inhibition with an IC_50_ value of 4.55 ± 0.13 μM. Molecular interaction analyses indicate that angustine binds within the EGCG-homologous region, where it is stabilized primarily through hydrophobic contacts and π–π stacking interactions involving residues such as Leu361 and Met47, thereby contributing to effective GUS inhibition [86]. Similarly, the phenylpropanoid cleomiscosin A is obtained from the seeds of *Cleome viscosa*, a plant widely distributed in tropical regions, where it accounts for approximately 0.2–0.3% of the seed content. This compound features a coumarin core with a furan substituent and functions as a mixed-type inhibitor of EcGUS (IC_50_ = 3.97 ± 0.35 μM). Its binding site is also localized within the EGCG-homologous region, with the coumarin ring participating in hydrophobic interactions with Phe161 and the furan hydroxyl group forming a hydrogen bond with Glu413, collectively contributing to GUS inhibition [80]. In addition, GUS inhibitory activity has also been observed among structurally distinct terpenoid natural products, further expanding the chemical space of microbial GUS modulators. Certain triterpenoid saponins have demonstrated GUS inhibitory potential. These compounds are extracted from the rhizomes of *Actinidia chinensis*, where triterpenoids comprise approximately 0.5–0.8% of the dry weight. A representative molecule, 2α,3α,24-trihydroxyurs-12-en-28-oic acid, is an ursane-type triterpenoid that contains hydroxyl groups at C-2α, C-3α, and C-24, along with a carboxyl group at C-28. This multi-hydroxyl/carboxyl configuration enables its function as a non-competitive inhibitor of EcGUS. Molecular docking studies reveal that the pentacyclic triterpenoid core inserts into an allosteric hydrophobic pocket formed by Asp203, Ser231, and Gly232. The 2α,3α-dihydroxyl groups form hydrogen bonds with Asp203, while the C-28 carboxyl group engages in a salt bridge with the amino group of Ser231. This combined binding mode—hydrophobic embedding reinforced by polar interactions—yields an inhibition constant (Kᵢ) of 12.8 μM [81].

Iridoid glycosides represent a less-explored class of natural GUS inhibitors. A repre-sentative compound, Symplolucidin B, is isolated from the fruits of Symplocos sumuntia, where it accounts for only 0.08–0.12% of the dry weight. It features a characteristic iridoid scaffold composed of a six-membered cyclic ether with an isoprenyl substituent and inhibits EcGUS with an IC_50_ of 17.2 ± 0.1 μM. Although its potency is one to two orders of magnitude lower than that of flavonoid- and tannin-based inhibitors, its dis-tinct molecular scaffold suggests a potentially unique mode of inhibition. However, the specific binding residues involved have yet to be characterized [83].

Compared with the extensive amount of plant-derived GUS inhibitors, fungal and bacterial metabolites represent a relatively limited but mechanistically informative subset. Classic examples include iminosugars from Streptomyces, such as nojirimycin and siastatin B, which mimic carbohydrate substrates or transition states and act as potent competitive GUS inhibitors, with optimized derivatives reaching nanomolar potency [9,87]. Beyond these early discoveries, relatively few new microbial GUS inhibitors have been reported in recent years. A representative 2024 study identified sorbicillinoid-type metabolites from the endophytic fungus Penicillium sp. HS-11, among which trichodimerol exhibited moderate inhibition toward EcGUS (IC_50_ = 92 ± 9.4 μM), likely by substrate-mimicking interactions within the catalytic pocket [84]. More recently, a 2025 investigation of fungi associated with medicinal hosts yielded a sesterterpenoid scaffold (e.g., versicolorin-type compounds) showing inhibitory activity comparable to the reference inhibitor saccharolactone (IC_50_ = 49.82 ± 1.21 μM), suggesting alternative non-flavonoid and non-phenolic frameworks for GUS modulation [85]. Overall, although microbial and fungal metabolites are numerically fewer than plant polyphenols, they provide structurally unique scaffolds and complementary inhibition modes that broaden the chemical space of GUS-targeted inhibitors.

### 4.2. Modulation of GUS Activity by Natural Product-Induced Microbial Changes

Gut microbial GUS activity is closely linked to the composition and balance of the intestinal microbiota, and dysbiosis can substantially alter both the abundance and function of these enzymes. For example, in irritable bowel syndrome (IBS), the loss of beneficial commensals such as *Alistipes* is associated with decreased fecal GUS activity, and diminished bilirubin deconjugation end-products, indicating a compromised microbial capacity for glucuronide hydrolysis [88]. Conversely, in recurrent Clostridioides difficile infection, overall microbial disruption diminishes GUS function, which can be restored by fecal microbiota transplantation, highlighting the sensitivity of GUS activity to microbial balance [89]. In colorectal cancer (CRC), gut microbiota dysbiosis is characterized not merely by taxonomic shifts but by a remodeling of the microbial GUS panoply, involving selective enrichment and depletion of specific GUS structural types. This functional reconfiguration is largely driven by alterations in GUS-producing Bacteroidota members—particularly *Bacteroides cellulosilyticus* [90]. Experimental animal models corroborate these findings, showing that induced microbial perturbations dynamically modulate GUS activity, and that targeted inhibition of GUS reduces xenobiotic reactivation while partially restoring microbial equilibrium [91]. Collectively, these observations indicate that both the composition and functional potential of gut microbiota dictate GUS activity, and that perturbations in microbial ecology translate directly into changes in glucuronide metabolism.

Current strategies to mitigate the adverse effects of gut microbial GUS have primarily focused on anti-inflammatory interventions or direct enzymatic inhibition, often neglecting the critical role of GUS-expressing bacteria (Figure 2B). A more comprehensive approach entails modulation of the microbial ecosystem to suppress GUS activity at its origin.

Chemotherapeutic agents such as CPT-11 disrupt gut microbial homeostasis by enriching pro-inflammatory and GUS-producing bacterial populations while depleting beneficial commensals [92]. Clinical and preclinical studies have demonstrated a robust association between CPT-11/SN-38-induced intestinal toxicity and the abundance of GUS-producing bacteria, particularly *Lactobacillus reuteri*. Fecal microbiota transplantation has demonstrated that colonization by *Lactobacillus reuteri* exacerbates intestinal inflammation and impairs epithelial differentiation, whereas its elimination alleviates colitis and preserves crypt stem cell function [66]. Additionally, Gao et al. reported that dehydrodiisoeugenol—a natural compound derived from nutmeg, ameliorates CPT-11-induced mucositis through selective suppression of GUS-producing *Enterococcus faecalis*, thereby reducing the conversion of SN-38G to toxic SN-38 and promoting intestinal stem cell proliferation and mucosal repair [93].

Beyond specific pathogen targeting, global modulation of gut microbial communities has emerged as a promising therapeutic strategy. The botanical drug YIV-906, derived from the classical Chinese formulation Huangqin Decoction, has shown efficacy in alleviating CPT-11-induced diarrhea [94,95]. Its multi-component composition acts synergistically: baicalin and baicalein preferentially inhibit pathogenic strains such as *Escherichia coli*, while paeoniflorin exerts stronger anti-inflammatory effects [96]. Similarly, Xiaochaihu Decoction has shown efficacy in alleviating CPT-11-induced diarrhea by enhancing beneficial microbial populations and reducing intestinal inflammation [16].

The role of GUS-producing bacteria extends well beyond chemotherapy induced toxicity to encompass other pathophysiological conditions. In a hyperglycemic mouse model, metagenomic analysis revealed that hyperglycemia fosters the dominance of GUS-producing bacteria, accompanied by elevated intestinal GUS activity. This enhancement promotes the hydrolysis of bile acid-glucuronide conjugates, resulting in excessive bile acid accumulation within both the serum and hepatic tissue. Subsequent activation of the ileal farnesoid X receptor suppresses glucagon-like peptide-1 (GLP-1) secretion from L-cells, thereby accelerating hyperglycemia progression. Treatment with vancomycin effectively eliminated GUS-producing bacteria, suppressed GUS activity and bile acid levels, restored GLP-1 secretion, and ultimately prevented hyperglycemia [97].

### 4.3. GUS-Mediated Biotransformation of Natural Products

Many glycoside compounds possess promising biological activities but are constrained as therapeutic agents because of their limited oral bioavailability [98]. A representative example is baicalin, the principal flavonoid in *Scutellaria baicalensis*, which consists of baicalein conjugated to glucuronic acid through a glycosidic linkage. Although baicalin exhibits a broad spectrum of pharmacological activities, including antioxidant, antimicrobial, anticancer, and hepatoprotective effects, it is subject to extensive first-pass metabolism in the gut following oral administration, leading to an absolute oral bioavailability as low as 2.2 ± 0.2% [99]. In contrast, its aglycone, baicalein, produced through hydrolysis of the glucuronide moiety, displays increased lipophilicity, facilitating passive transmembrane diffusion. This structural transformation substantially enhances both systemic bioavailability and pharmacological efficacy. For instance, in osteosarcoma cell models, baicalein exhibited markedly stronger, dose-dependent antiproliferative effects than baicalin [100].

A similar metabolic pathway is observed for glycyrrhizin, a triterpenoid saponin containing two glucuronic acid moieties. Sequential hydrolysis by GUS first produces glycyrrhetinic acid monoglucuronide, followed by glycyrrhetinic acid. Although glycyrrhizin is used clinically, its application is limited by dose-dependent side effects, prompting structural modification strategies to enhance its therapeutic profile [101]. The resulting monoglucuronide, formed via cleavage of one β-1,2-glycosidic bond, exhibits intermediate polarity that facilitates membrane permeation and intestinal absorption, resulting in improved pharmacokinetic and pharmacological properties over the parent compound. As a sweetener, it is significantly sweeter than glycyrrhizin and exhibits enhanced solubility, improved taste, and reduced toxicity. These advantages, along with enhanced antitumor activity, position it as a promising candidate for anticancer drug development [102]. The fully hydrolyzed product, glycyrrhetinic acid, is highly lipophilic and cannot readily cross cellular membranes via passive diffusion. Nevertheless, as the ultimate bioactive metabolite of glycyrrhizin, it retains potent pharmacological activity even at low doses. Afkhami-Poostchi et al. employed a GUS-overexpressing *E. coli* strain to locally convert glycyrrhizin into glycyrrhetinic acid in proximity to colon cancer cells, significantly suppressing tumor growth [103].

Upon liberation, bioactive aglycones such as baicalein and glycyrrhetinic acid may be reconjugated by hepatic UGT enzymes upon entry into systemic circulation. These conjugates are then excreted into the intestine via bile, where gut microbial GUS regenerates the active aglycones. This forms a cyclic “activation–circulation–reactivation” process that extends their *in vivo* residence time and enhances overall bioavailability (Figure 2C). Thus, gut microbial GUS acts as a critical enzymatic hub in modulating the pharmacological effectiveness of plant-derived glycosides.

Notably, GUS also exerts site-specific regulatory functions within inflammatory microenvironments. Studies indicate that at lipopolysaccharide induced sites of inflammation, recruited neutrophils secrete GUS, which locally hydrolyzes luteolin monoglucuronide to regenerate free luteolin aglycone. This localized activation concentrates anti-inflammatory effects precisely at inflamed tissues, enhancing therapeutic specificity while minimizing systemic toxicity [104]. Together, these findings underscore the role of GUS as a central metabolic nexus governing the site-specific bioavailability and efficacy of numerous natural bioactive compounds.

## 5. Natural Product Interactions with GUS in Health and Disease

GUS functions as a pivotal modulator of drug metabolism and intestinal homeostasis, with its dysregulation increasingly implicated in various pathologies, most notably chemotherapy-induced intestinal injury. High throughput screening using recombinant EcGUS has identified numerous natural inhibitors, including flavonoids, alkaloids, and polyphenols, thereby broadening the chemical repertoire for targeted interventions and facilitating preliminary structure–activity relationship analyses [105]. However, the field remains limited by its dependence on in vitro enzyme inhibition assays and computational docking, which, although valuable for characterizing direct binding, offer minimal insight into in vivo efficacy or biological specificity. In physiological contexts, factors such as isoform diversity, host–microbiome interactions, systemic pharmacokinetics, and tissue-level microenvironments significantly influence inhibitor performance. As a result, the translational relevance of most in vitro identified GUS inhibitors remains largely unverified, underscoring the need for functional validation in disease-relevant models.

Nonetheless, targeted investigations have yielded promising advances. In a systematic study of MMF-induced enteropathy, octyl gallate was identified from a natural product library of 2115 compounds as a potent and selective GUS inhibitor. In vivo assessments demonstrated its capacity to suppress intestinal GUS activity, reduce local accumulation of toxic MPA metabolites, and alleviate gastrointestinal injury, highlighting its therapeutic potential for mitigating drug-induced gut toxicity [106].

Tea-derived polyphenols also exemplify a dual-targeting approach to GUS modulation. In a rat model of CPT-11-induced delayed-onset diarrhea, green tea extract significantly reduced symptom severity by directly inhibiting GUS enzymatic activity and concurrently suppressing GUS-producing bacterial populations—including *Bacteroides fragilis*, *Escherichia coli*, and *Clostridium perfringens*. This combined effect effectively limited intestinal SN-38 exposure and prevented mucosal damage [107].

Additional support comes from natural agents such as berberine and the herbal formula Huangqin Decoction, both of which selectively inhibit GUS in vivo. These interventions disrupt the intestinal hydrolysis of CPT-11 metabolites, reducing SN-38 regeneration and accumulation in colonic tissue, thereby alleviating gastrointestinal toxicity [96,108,109]. Collectively, these findings underscore the feasibility of using natural products to modulate GUS activity and highlight the necessity of validating such effects under physiologically and pathologically relevant conditions.

## 6. Conclusions and Perspectives

This review systematically outlines the structural features and phylogenetic classification of GUS, along with its central regulatory roles in host physiology and pathophysiology through the metabolism of both endogenous compounds and xenobiotics. Given the pivotal role of GUS in drug-induced toxicity, natural products with multi-target modulatory properties have emerged as promising candidates for therapeutic intervention. Recent studies have identified a variety of natural products capable of directly inhibiting GUS enzymatic activity and indirectly modulating its expression by reshaping gut microbiota composition. The feasibility of mitigating specific disease phenotypes via targeted GUS modulation has been preliminarily demonstrated in preclinical models.

Despite these advances, several critical challenges remain in this field. Most current studies remain largely descriptive, focusing on gross changes in GUS activity or microbial community structure following natural product intervention, while lacking mechanistic insight at the molecular level. It is imperative to integrate advanced omics technologies—such as metagenomics, metatranscriptomics, and metaproteomics—to precisely identify microbial taxa responsible for functional GUS expression in disease states and to systematically dissect transcriptional and translational regulatory networks, thereby elucidating the dynamic responses of functional GUS-producing microbiota and their molecular targets to natural product interventions.

Current research systems face additional obstacles, including interspecies variation in gut microbiota composition and GUS isoform profiles, which significantly impede the translational applicability of preclinical findings. Moreover, existing studies rely heavily on in vitro screening and mechanistic inference, often lacking robust in vivo validation within complex biological contexts. Future studies should prioritize the development of refined in vitro and in vivo evaluation platforms and employ multi-dimensional validation strategies to elucidate the molecular mechanisms underlying GUS-related disease pathogenesis. Systematic development and functional validation of GUS modulators are strongly recommended to deepen mechanistic understanding of GUS biology and to enable the design of targeted intervention strategies for GUS associated diseases.

## Figures and Tables

**Table 1:** Structural classes and phylum specificity of GUS.

GUS Categories	Description	Abundance (%)	Representative Sources	Membrane Traversal	References
Loop 1	Initially termed the “Bacterial Loop” in EcGUS, >15 residues	5.5% (human) 8.6% (mouse)	*Bacillota*	Intracellular	[21,22]
Mini-Loop 1	Located in the same position as L1, 10–15 residues	15–21% (human) 6.5% (mouse)	*Bacillota* *Bacteroidota*	Secreted by *Bacteroidota*: Yes Secreted by *Bacillota*: No	[21,22]
Loop 2	Adjacent in structure to L1, a full size of ≥12 residues	12–14% (human) 6.8% (mouse)	*Bacillota* *Bacteroidota*	Able to traverse the inner microbial membrane	[21,22]
Mini-Loop 2	Located in the same position as Mini-Loop 2, 9–11 residues	4–7% (human) 15.3% (mouse)	*Bacteroidota* *Verrucomicrobiota*	Able to traverse the inner microbial membrane	[21,22]
Mini-Loop 1,2	Contain both mL1 and mL2 motifs, exhibit 10–15 residues in the L1 and 9–12 residues in L2.	1–2%	*Verrucomicrobiota* *Bacteroidota*	Able to traverse the inner microbial membrane	[21,22]
NL	NL GUS enzymes lack a loop in either position	49–57%	*Bacillota* *Bacteroidota*	Secreted by *Bacteroidota*: Yes Secreted by *Bacillota*: No	[21,22]
FMN	FMN-binding glycoside hydrolase	-	*Bacillota*	-	[23]

**Table 2:** Direct inhibition of gut microbial GUS by different classes of natural products.

Compound Class	Compound Name	Structure	GUS Source	IC_50_/K_i_ (μM)	Inhibition Type	Binding Interactions/Binding Site	Substrate/Positive Control	Reference
	2′-Hydroxychrysin	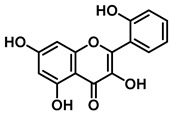	EcGUS	3.16 ± 0.34/3.07	Non-competitive	Hydrophobic and polar interactions/Phe554, Phe448, His162, Asp163, Tyr472, Glu413	PNPG/ EGCG	[38]
	Jaceosidin	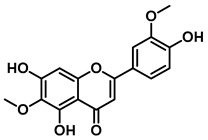	EcGUS	5.70 ± 0.08/6.18	Mixed	Hydrophobic and polar interactions/Met447, Leu361, Phe448, Asn566, Trp549, His330, Asp163, Asn412, Glu413, Tyr468	PNPG/ EGCG	[38]
	5,7,4′,5′-Tetrahydroxy-6,3′-dimethoxyflavone	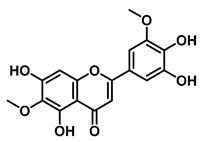	EcGUS	3.82 ± 0.10/3.55	Mixed	Hydrogen bonding/Lys568, Trp549, Asn566, Met447	PNPG/ EGCG	[38]
	3′-Geranyl genistein	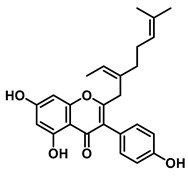	EcGUS	3.65 ± 0.28/–	Mixed	Hydrogen bonding and hydrophobic interactions/Glu413, Glu504, Phe161, His162, Asp163, Tyr472, Met447	PNPG/ EGCG	[38]
	3′-Geranyl-5,7,2′,4′-tetrahydroxyisoflavone	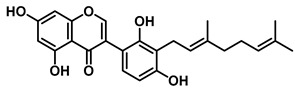	EcGUS	3.97 ± 0.11/–	Non-competitive	Hydrogen bonding and hydrophobic interactions/Glu413, Met447, Tyr472, Arg562	PNPG/ EGCG	[38]
	Myricetin	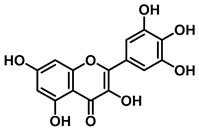	EcGUS	3.95 ± 0.04/3.07	Non-competitive	Hydrogen bonding and electrostatic interactions/Glu504, Glu413, Trp549	PNPG/ EGCG	[69]
	Chrysoeriol	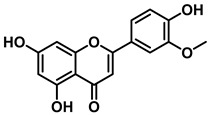	EcGUS	4.94 ± 0.11/4.58	Non-competitive	Hydrophobic interactions/Leu361, Val355, Val446, Met447	PNPG/ EGCG	[69]
	Amentoflavone	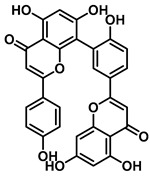	EcGUS	0.62 ± 0.072 (DDAOG)/– 0.49 ± 0.03 (SN-38G)/–	Mixed (DDAOG) Competitive (SN-38G)	Hydrogen bonding/Leu361, Ile363, Glu413	DDAOG/SN38G	[70]
	Scutellarein	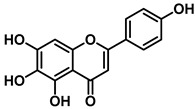	EcGUS	5.76 ± 1.53/–	Competitive	Hydrogen bonding/Glu413, Arg562	PNPG/ DSL	[71]
	Luteolin	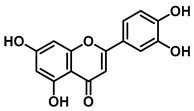	EcGUS	8.68 ± 2.02/–	Competitive	Hydrogen bonding/Glu413, Arg562	PNPG/ DSL	[71]
	Steppogenin	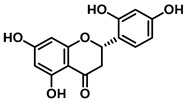	EcGUS	6.21 ± 0.20/–	Mixed	Hydrophobic interactions and hydrogen bonding/Phe161, Phe448, Glu413, Tyr472	PNPG/ EGCG	[72]
	Azaleatin	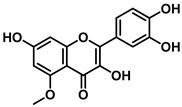	EcGUS	0.57 ± 0.04/–	Non-competitive	Hydrophobic interactions and hydrogen bonding/Phe448, Tyr468, Glu413	PNPG/ EGCG	[73]
	Morin	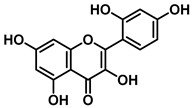	EcGUS	1.12 ± 0.09/–	Non-competitive	Hydrogen bonding/Tyr160, Gln158, Lys157, His162, Ser159, Ser557, Ile560	PNPG/ DSL	[74]
	Sanggenon C	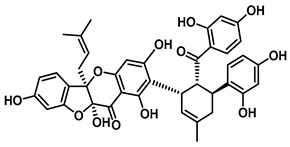	EcGUS	2.07 ± 0.06/–	Non-competitive	Hydrogen bonding and pi-alkyl interactions/Ser159, Ser557, Tyr160, Lys157, Leu361, Ile149	PNPG/ DSL	[74]
	Kuwanon G	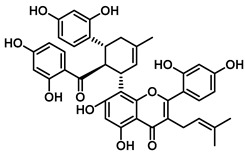	EcGUS	2.37 ± 0.11/–	Non-competitive	Hydrophobic interactions and hydrogen bonding/Ser159, Tyr160, His162, Asp163, Lys157, Gln158, Leu361, Ile149, Ser557, Ile560	PNPG/ DSL	[74]
	Sanggenol A	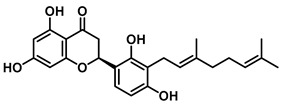	EcGUS	3.27 ± 0.17/–	Non-competitive	Hydrophobic interactions and hydrogen bonding/Ser159, Tyr160, His162, Asp163, Lys157, Gln158, Leu361, Ile149, Ser557, Ile560	PNPG/ DSL	[74]
	Kuwanon C	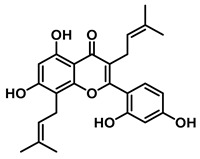	EcGUS	4.27 ± 0.32/–	Non-competitive	Hydrophobic interactions and hydrogen bonding/Ser159, Tyr160, His162, Asp163, Lys157, Gln158, Leu361, Ile149, Ser557, Ile560	PNPG/ DSL	[74]
	Amentoflavone	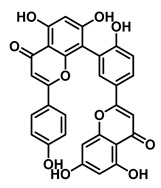	*C. perfringens* (CpGUS)	2.36 ± 0.12/–	Non-competitive	Hydrogen bonding and pi-alkyl interactions/Arg574, Lys315, Glu55, Ala556, Lys577	PNPG/ DSL	[75]
			*S. pasteuri* (SpasGUS)	2.88 ± 0.38/–	Non-competitive	Hydrogen bonding and pi-alkyl interactions/His336, Glu508, Asn414, His364, Ile565, Arg566	PNPG/ DSL	[75]
			EcGUS	3.43 ± 0.32/–	Non-competitive	Hydrogen bonding/Asp163, Ser557, Trp160, Lys157	PNPG/ DSL	[75]
	Moricitrin B	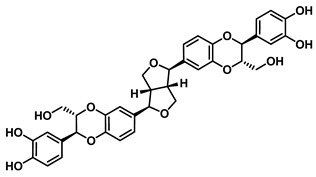	EcGUS	0.95 ± 0.01/–	Not specified	–/–	4-MUG/ DSL	[76]
	Americanol B	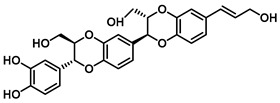	EcGUS	4.02 ± 0.06/–	Not specified	–/–	4-MUG/ DSL	[76]
	Isoamericanol B	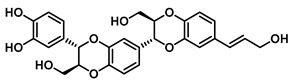	EcGUS	6.91 ± 0.16/–	Not specified	–/–	4-MUG/ DSL	[76]
Natural Amides	Grossamide	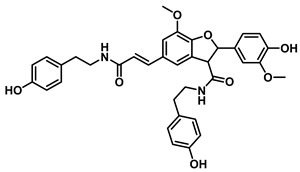	EcGUS	0.73 ± 0.03/–	Non-competitive	Electrostatic and hydrophobic Interactions/Glu413, Glu504, Arg562	PNPG/ EGCG	[72]
	Grossamide K	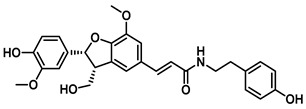	EcGUS	1.24 ± 0.06/–	Non-competitive	Hydrogen bonding and hydrophobic interactions/Ser159, Asn358, Leu561, Arg562	PNPG/ EGCG	[72]
	N-Feruloyltyramine	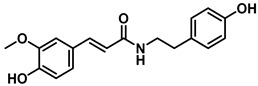	EcGUS	12.16 ± 0.7/–	Not specified	Hydrogen bonding/Asp163, His520, Arg562	PNPG/ EGCG	[72]
	Piperlongumamide F	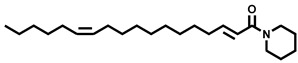	EcGUS	4.9 ± 0.9/3.1	Non-competitive	Van der waals interactions and hydrogen bonding/Trp340, Ala316	PNPG/ DSL	[77]
	1-(Eicosa-2E,14Z-dienoyl)piperidine	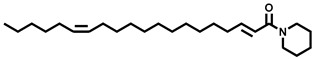	EcGUS	8.1 ± 0.7/12.8	Non-competitive	Hydrogen bonding and hydrophobic interactions/Val312, Asp319	PNPG/ DSL	[77]
	N-Isobutyl-(2E,4E)-undeca-2,4-dienamide	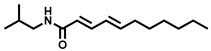	EcGUS	5.8 ± 1.2/8.8	Uncompetitive	Hydrophobic interactions/Tyr160, Leu361, Tyr472	PNPG/ DSL	[77]
	(2E,4E,12Z)-N-Isobutyloctadeca-2,4,12-trienamide)	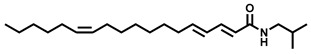	EcGUS	6.3 ± 0.9/5.3	Uncompetitive	Alkyl interactions/Ile363, Phe365	PNPG/ DSL	[77]
	(2E,4E,14Z)-N-Isobutyleicosa-2,4,14-trienamide	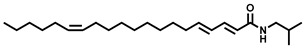	EcGUS	6.2 ± 2.4/8.8	Uncompetitive	Van der waals interactions and hydrophobic interactions/Tyr160, His162, Asp163	PNPG/ DSL	[77]
	(2E,4E,16Z)-N-Isobutyldocosa-2,4,16-trienamide	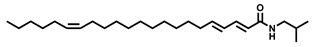	EcGUS	5.1 ± 1.5/9.0	Uncompetitive	Van der waals interactions and hydrophobic interactions/Tyr160, Gln158, Phe161	PNPG/ DSL	[77]
Tannin	1,2,3,4,6-Penta-O-galloyl-β-D-glucopyranose (PGG)	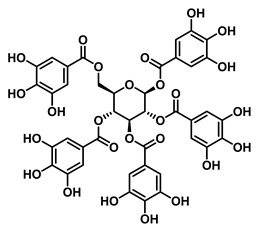	EcGUS	0.13 ± 0.004/0.30	Non-competitive	Hydrogen bonding/Glu106, His107, Glu108, Gly109	4-MUG/ DSL	[78]
			CpGUS	0.19 ± 0.001/0.17	Non-competitive	Hydrogen bonding/Lys147, Val161, Asp164, Met364	4-MUG/ DSL	[78]
			*Streptococcus agalactiae* (SaGUS)	0.14 ± 0.003/0.12	Non-competitive	Hydrogen bonding/Asp46, Asp550, Glu552, Glu561	4-MUG/ DSL	[78]
			*Eubacterium eligens* (EeGUS)	0.55 ± 0.003/1.29	Non-competitive	Hydrogen bonding/Asp58, His62, Tyr63, Asn134	4-MUG/ DSL	[78]
Catechins And Theaflavins	Catechin gallate	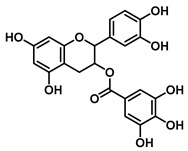	EcGUS	1.48/–	Mixed	Hydrogen bonding and hydrophobic interactions/Glu413, Ser360, Ile560	DDAOG/-	[79]
	Theaflavin-3′ monogallate	* 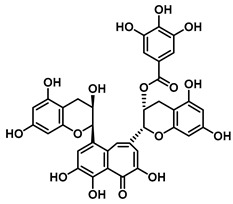 *	EcGUS	1.03/–	Mixed	Hydrogen bonding/Leu359, Asn369, Arg417	DDAOG/-	[79]
	Theaflavin-3,3′-digallate	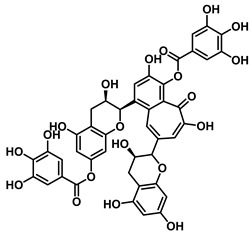	EcGUS	1.41/–	Mixed	Hydrogen bonding/Ser360, Glu413, Ile560	DDAOG/-	[79]
Alkaloids	Angustine	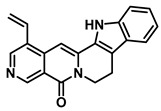	EcGUS	4.55 ± 0.13/–	Non-competitive	Hydrophobic interactions and π-π stacking/Leu361, Met47	PNPG/ EGCG	[72]
Phenylpropanoids	Cleomiscosin A	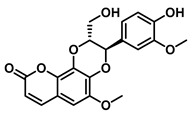	EcGUS	3.97 ± 0.35/–	Mixed	Hydrophobic interactions and hydrogen bonding/Phe161, Asp163, Tyr472, Arg562	PNPG/ EGCG	[80]
Quinones	Mansonone H	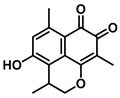	EcGUS	10.32 ± 1.85/–	Non-competitive	Hydrophobic interactions and polar interaction/Glu413, Leu361, Val446, Met447	PNPG/ EGCG	[80]
Terpenoids	2α,3α,24-trihydroxyurs-12-en-28-oic acid	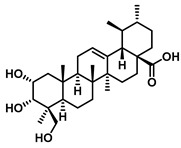	EcGUS	14.36 ± 0.42/12.8	Non-competitive	Hydrogen bonding and salt bridge/Asp203, Ser231, Gly232	PNPG/ DSL	[81]
	Hemslonin B	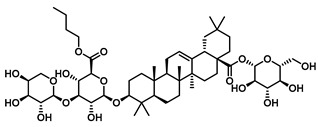	EcGUS	1.0 ± 0.1/–	Not specified	–/–	PNPG/ DSL	[82]
	Momordin IIa	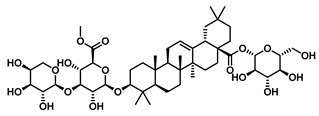	EcGUS	8.3 ± 0.3/–	Not specified	–/–	PNPG/ DSL	[82]
	3β-hydroxyolean-12-en-28-oic acid-28-O-β-D-glucopyranoside	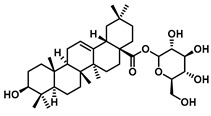	EcGUS	14.0 ± 1.3/–	Not specified	–/–	PNPG/ DSL	[82]
	Symplolucidin B	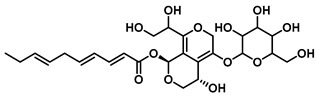	EcGUS	17.2 ± 0.1/–	Not specified	–/–	PNPG/ DSL	[83]
Sorbicillinoids	Trichodimerol	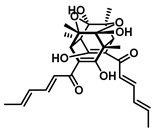	EcGUS	92.0 ± 9.4/–	Not specified	–/–	PNPG/ DSL	[84]
Sesterterpenoids	Versicolorin A	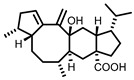	EcGUS	49.82 ± 1.21/–	Not specified	–/–	PNPG/ DSL	[85]

Abbreviations: PNPG, p-nitrophenyl-β-D-glucuronide; DDAOG, 9H-(1,3-dichloro-9,9-dimethylacridin-2-one-7-yl) β-D-glucuronide; 4-MUG, 4-methylumbelliferyl β-D-glucuronide; SN-38G, SN-38 β-D-glucuronide; DSL, D-saccharic acid 1,4-lactone.

## Data Availability

Data sharing not applicable to this article as no datasets were generated or analyzed during the current study.
